# Purified Human Pancreatic Duct Cell Culture Conditions Defined by Serum-Free High-Content Growth Factor Screening

**DOI:** 10.1371/journal.pone.0033999

**Published:** 2012-03-19

**Authors:** Corinne A. Hoesli, James D. Johnson, James M. Piret

**Affiliations:** 1 Michael Smith Laboratories and Department of Biological and Chemical Engineering, University of British Columbia, Vancouver, Canada; 2 Department of Surgery, University of British Columbia, Vancouver, Canada; 3 Department of Cellular and Physiological Sciences, University of British Columbia, Vancouver, Canada; University of Bremen, Germany

## Abstract

The proliferation of pancreatic duct-like CK19+ cells has implications for multiple disease states including pancreatic cancer and diabetes mellitus. The in vitro study of this important cell type has been hampered by their limited expansion compared to fibroblast-like vimentin+ cells that overgrow primary cultures. We aimed to develop a screening platform for duct cell mitogens after depletion of the vimentin+ population. The CD90 cell surface marker was used to remove the vimentin+ cells from islet-depleted human pancreas cell cultures by magnetic-activated cell sorting. Cell sorting decreased CD90+ cell contamination of the cultures from 34±20% to 1.3±0.6%, yielding purified CK19+ cultures with epithelial morphology. A full-factorial experimental design was then applied to test the mitogenic effects of bFGF, EGF, HGF, KGF and VEGF. After 6 days in test conditions, the cells were labelled with BrdU, stained and analyzed by high-throughput imaging. This screening assay confirmed the expected mitogenic effects of bFGF, EGF, HGF and KGF on CK19+ cells and additionally revealed interactions between these factors and VEGF. A serum-free medium containing bFGF, EGF, HGF and KGF led to CK19+ cell expansion comparable to the addition of 10% serum. The methods developed in this work should advance pancreatic cancer and diabetes research by providing effective cell culture and high-throughput screening platforms to study purified primary pancreatic CK19+ cells.

## Introduction

The pancreas is a complex organ containing multiple interspersed cell types with diverse exocrine and endocrine functions. Established protocols exist for isolating, studying and transplanting relatively pure cultures of endocrine islet cells [1], [2], [3], but the culture of enriched CK19-positive ductal cells has proven challenging. Human CK19-positive cells are an interesting cell population [4] as they have been shown to proliferate in several disease states, including pancreatic ductal adenocarcinoma [5] and diabetes [6]. Some groups have also suggested that cells with a ductal phenotype, potentially centroacinar cells [7], have the potential to express measurable amounts of insulin *in vitro* [8], [9], [10], [11], [12] or *in vivo* after pancreas injury [12], [13]. However, the results from lineage tracing experiments that could prove the conversion of duct cells to β-cells have mostly been negative [14], [15], [16], [17]. Multiple groups have tried to maintain human duct cells in long-term culture [10], [18], [19], but rapidly dividing vimentin-positive cells quickly overgrow adherent cultures of mixed exocrine cells [18], [20]. In addition, most pancreatic duct cell culture protocols require the use of serum-containing medium, which is undesirable for research or therapeutic cell culture because serum is undefined, suffers from high batch-to-batch variation and may contain dangerous contaminants that are problematic for transplantation applications [21], [22]. Improved methods to separate exocrine cell populations and to culture them in defined optimized media are needed to enable studies of duct cell biology.

One reported method to obtain 93∼97% pure CK19+ cell cultures isolated pancreatic cells expressing the carbohydrate antigen 19-9 (Ca19-9) [23]. However, the cell phenotypes obtained after 7 days of culture were not quantified and the Ca19-9 enriched cells expanded poorly [24]. Since vimentin-positive cells are the main competing population, an alternative approach would be to selectively remove this population. This negative cell sorting strategy would have the advantage of leaving the cells of interest free from surface antibodies and magnetic beads that may affect cell behaviour [25], [26]. The *in vitro* differentiation potential of fibroblast-like cells expanded from adult human pancreatic tissue resembles the differentiation potential expected for mesenchymal stem cells, expressing markers including CD90 and capable of chondrogenic or osteogenic differentiation [20]. CD90.1-positive cells enriched from rat pancreatic tissue adopt a fibroblast-like phenotype in culture and express much lower levels of duct markers than CD90.1-depleted cells [27].

We present a novel method to obtain purified human CK19-positive cell cultures from mixed pancreatic exocrine tissue and to quantify their proliferation in a high-throughput assay. Depletion of CD90-expressing cells successfully eliminated most fibroblast-like cells from the cultures. High-throughput imaging of the CD90-depleted cultures in 96-well plates allowed the multifactorial testing of the effects of five reported duct mitogens on apoptosis, expansion and proliferation. This led to the development of a serum-free medium to replace the use of serum and provide defined culture conditions for pancreatic duct cell culture research.

## Methods

### Cell culture

Islet-depleted pancreatic cell aggregates from human donors (10 in total; age 39±19 years; body weight 72±13 kg) were kindly provided by Dr. Garth Warnock and Dr. Ziliang Ao at the Ike Barber Human Islet Transplant Laboratory (Vancouver, BC, Canada). Pancreata were obtained with the written informed consent of family members under the approval of the University of British Columbia Clinical Research Ethics Board. Cell clusters received on day 0 were washed twice in CMRL medium with 10% fetal bovine serum, 100 units/mL penicillin and 100 µg/mL streptomycin (all from Invitrogen, Carlsbad, CA), referred to as CMRL+10% FBS medium. Clusters were then dispersed or seeded overnight in CMRL+10% FBS medium at 1 µL packed cell volume (PCV)/cm^2^ (PCV tubes, Techno Plastic Product, Trasadingen, Switzerland) in tissue culture-treated flasks (Sarstedt, Nümbrecht, Germany) before magnetic-activated cell sorting (MACS) on day 1. Unsorted or sorted cells were seeded at 1.25×10^5^ cells/cm^2^ in 0.32 mL/cm^2^ CMRL+10% FBS medium in plates containing 0.32 mL/cm^2^ pre-incubated medium and cultured at 37°C, 5% CO_2_ and 90% humidity. Live cells were enumerated by trypan blue exclusion using a Cedex cell counter (Roche Innovatis, Bielefeld, Germany). The unsorted and MACS-sorted cultures were maintained in CMRL+10% FBS medium for 8 days, with medium exchanges on days 2, 5 and 7. For serum-free assay development and screening, the cultures were shortened to 6 days with medium exchanges on days 2 and 5. The basal CMRL-0.1X ITS serum-free medium consisted of CMRL with 0.5 mg/L insulin+0.5 mg/L transferrin+0.5 µg/L selenite (i.e. 0.1 times I-1884 from Sigma), 10 mM nicotinamide and 0.2% BSA (STEMCELL, BC, Canada). The test solutions contained 0.1%, 1% or 10% FBS (Invitrogen), or 50% pancreatic fibroblast-conditioned medium diluted in CMRL-0.1X ITS. The following recombinant human growth factors were also tested at 20 ng/mL unless otherwise mentioned: basic fibroblast growth factor (bFGF, STEMCELL), epidermal growth factor (EGF, STEMCELL), hepatocyte growth factor (HGF, Sigma), keratinocyte growth factor (KGF, Sigma), vascular endothelial growth factor (VEGF, Sigma). One h after the last medium exchange, 10 µM of 5-Bromo-2′-deoxy-uridine was added (BrdU, Labelling and Detection Kit II, Roche, Basel, Switzerland) and incubated for 20 h prior to fixing, staining and Cellomics analysis.

### Conditioned medium

Passaged (P3 to P8) CD90-enriched pancreatic cells cultured in CMRL+10% FBS medium until reaching 90% confluency (∼6×10^4^ cells/cm^2^) were treated with 10 µg/mL mitomycin c (Sigma) for 1 h, washed 3 times and incubated for 24 h in CMRL-0.1X ITS, then filter-sterilized and stored at −80°C.

### Cell dispersion

Cell clusters were washed twice with dispersion medium (1 mM EDTA from Invitrogen, 10 mM HEPES from Sigma and 0.5% bovine serum albumin from STEMCELL prepared in Ca^2+^ and Mg^2+^-free HBSS from Invitrogen), then re-suspended at 0.05 mL PCV/mL in dispersion medium and kept at 37°C for 7 min with 75 rpm agitation. Clusters were digested for 10 min at 37°C by adding 25 µg/mL trypsin and 4 µg/mL DNase (Sigma). After adding CMRL+10% FBS medium, the cells were triturated and filtered through a 40 µm nylon sieve (BD Biosciences). The total cell yield based on PCV was 43±7%, providing 168±39 million live dispersed cells/mL PCV initial tissue clusters.

### Magnetic-activated cell sorting

Adherent cells from undispersed clusters were washed with MACS buffer consisting of Ca^2+^ and Mg^2+^-free phosphate buffered saline (PBS) with 1% FBS, 2 mM EDTA, 100 units/mL penicillin and 100 µg/mL streptomycin. Cells were trypsinized, triturated after adding CMRL+10% FBS medium and then filtered through a 40 µm cell strainer (BD Biosciences), yielding 0.17±0.05×10^6^ cells/cm^2^ on day 1. The cells were then washed twice with MACS buffer and re-suspended at ≤2×10^7^ cells/mL in ≥50 µL MACS buffer. An equal volume of primary antibody was added to obtain 1∶100 mouse anti-Ca19-9 (#NCL-L-CA19-9, Leica Microsystems, Germany) or 1∶500 mouse anti-CD90 (#555593, BD Pharmingen, CA). After incubating for 25 min on ice at 75 rpm agitation, the cells were washed twice with MACS buffer. Magnetic labeling was performed using microbead-labelled goat or rat anti-mouse IgG1 antibody (#130-048-402 or #130-047-102, Miltenyi Biotec, Germany). Cells were re-suspended at 4×10^6^ cells/mL and separated using the “possel” (for Ca19-9) or “depletes” (for CD90) program of an Automacs® (Miltenyi) cell separator. The total live cell yield from MACS was 60±3%.

### Flow cytometry

Adherent cells were washed with PBS, trypsinized, triturated after adding FACS buffer (PBS+10% FBS) and filtered through a 40 µm strainer. Cells kept on ice were then washed twice with FACS buffer, distributed to obtain 2.5×10^5^ cells/sample, centrifuged and re-suspended in 50 µL. Then, 50 µL of primary antibody diluted in FACS buffer (same concentrations as for MACS) were added, followed by 25 min incubation at 75 rpm. The cells were washed twice, re-suspended in 50 µL and 50 µL of Alexa 647 labelled goat anti-mouse IgG (A21450, Invitrogen) were added to obtain a 1∶200 dilution. After 15 min incubation in the dark at 75 rpm, 10 µL of propidium iodide at 100 µg/mL (Sigma, in PBS) was added. The cells were washed twice, re-suspended in 500 µL FACS buffer and analyzed on a BD FACSCalibur flow cytometer. Data analysis including compensation was performed with Flowjo 7.2.5 software (Tree Star, Ashland, OR).

### Immunocytochemistry and Cellomics

Cell fixing and staining with BrdU was performed according to the BrdU kit instructions (Roche), except for diluting primary and secondary antibodies 1∶20. For samples that did not require BrdU labeling, the cells were fixed with Bouin's fixative for 15 min and stored in 70% ethanol. CK19 or Ki67 antigen retrieval was performed by microwaving 6 times for 5 s at 1000 W in 10 mM citrate (Sigma) at pH 6.0 and cells permeabilized by a 10 min incubation in 0.25% Triton X 100 (Sigma) dissolved in PBS. Unless otherwise mentioned, all subsequent incubations were at room temperature and 100 rpm agitation. All samples were then washed with PBS, incubated in blocking solution (Dakocyotmation, Glostrup, Denmark) for 15 min and then stained overnight at 4°C with primary antibodies in Antibody Diluent (Dako). Primary antibodies were 1∶200 guinea pig anti-human insulin (Dako A0564), 1∶1000 rabbit anti-human amylase (Sigma A8273), 1∶200 mouse anti-human CK19 (Dako M0888), 1∶200 mouse anti-human vimentin (Dako M0725), 1∶50 rabbit anti-human Ki67 (Santa Cruz Biotech sc-15402, Santa Cruz, CA) and/or 1∶200 mouse anti-human Ki67 (BD Biosciences 556003). The next day, cells were washed with PBS and stained 1 h in the dark with secondary antibodies (Alexa 488 goat anti-guinea pig IgG, Alexa 488 or 568 goat anti-rabbit IgG and/or Alexa 568 goat anti-mouse IgG, all from Invitrogen) at 1∶200 in Antibody Diluent. Samples were then washed, stained with 1 µg/mL DAPI for 15 min, and washed again. The plates were imaged on a Cellomics ArrayScan VTI. Slides were mounted with Vectashield medium (Vector Labs, Burlingame, CA) and imaged on a Zeiss Axioplan 2 microscope (Carl Zeiss, Oberkochen, Germany), using ImageJ analysis software (NIH).

### Design of experiments and statistical analysis

Results represent the average values obtained from 3 to 5 pancreata ± standard error of the mean. Two-way comparisons were based on Student's t-tests with p-values<0.05 considered significant, with a paired t-test used in the case of the comparison between the CK19+ cell number and BrdU incorporation read-outs. For comparison between a basal response and responses normalized to the basal response, confidence intervals with α levels of 0.05 were used. The main and interaction effects of bFGF, EGF, HGF, KGF and VEGF were quantified by a two-level (low level 0 ng/mL; high level 20 ng/mL) full factorial design with 8 centre points (10 ng/mL of all five factors). For each pancreas, these 40 conditions were repeated on three 96-well plates with different randomization on each plate. The results were analyzed with JMP 7.0 or 8.0 statistics software (SAS, Cary, NC). The growth factor concentrations *C_i_* were transformed into scaled variables 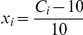. The model was as follows: 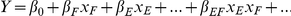 up to the fifth-order interaction effect 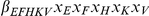, where *β_i_* values represent the fitted model parameters. The subscripts F, E, H, K, V represent the “*i*” factors bFGF, EGF, HGF, KGF and VEGF. The model was then reduced to exclude factors with p-values>0.1.

## Results

### Cultures of human exocrine pancreatic tissue give rise to duct-like epithelial cells and fibroblast-like cells

**Figure 1:**
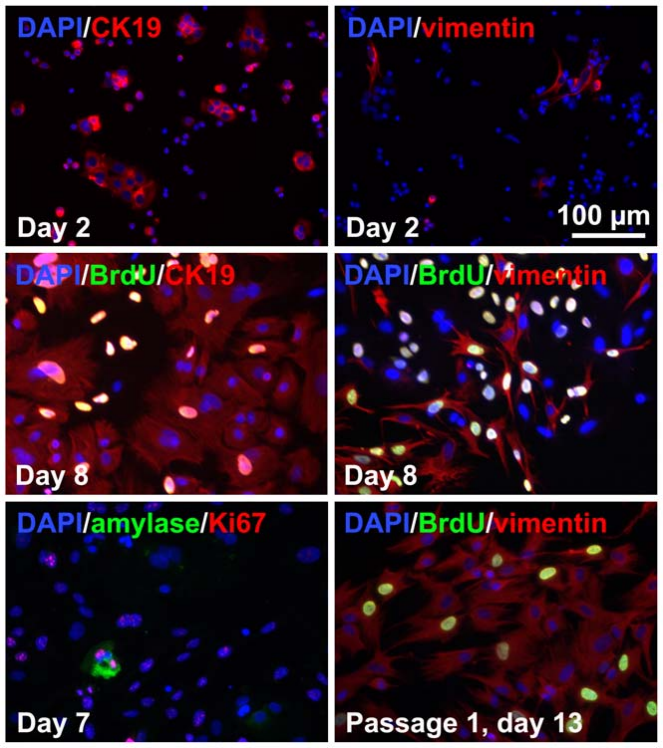
Phenotype of dispersed unsorted islet-depleted pancreatic cells cultured in serum-containing medium. On day 8, mixed cultures of CK19+ duct-like cells and vimentin+ fibroblast-like cells with very rare amylase+ cells are obtained. After a single passage, cultures consisted mainly of vimentin+ cells.

The cell types in islet-depleted cell clusters and derived adherent cultures were assessed. The initial dispersed tissue (day 0) consisted of 61±16% amylase+ cells, 6±3% CK19+ cells, 12±6% vimentin+ cells and 0.04±0.01% insulin+ cells. The fraction of insulin+ cells remained <1% throughout the culture period. After 2 days of adherent culture, the total adherent cell number was 27±16% of the cell number seeded, as expected for cultures containing significant numbers of human acinar exocrine cells [28]. Most CK19+ cells were in small clusters by day 2 (Figure 1), suggesting that these cells proliferated within 2 days, were incompletely dispersed or re-aggregated selectively. On the contrary, the vimentin+ cells were dispersed as single cells. At day 8, the cultures mainly consisted of duct-like cells in rounded cobblestone patterns surrounded by fibroblast-like spindle-shaped cells. The remaining amylase+ cells accounted for <1% of the cells and appeared unhealthy. Both main populations were proliferative at day 7 (44±26% BrdU+ cells among CK19+ cells; 60±24% BrdU+ cells among vimentin+ cells). After a single passage (day 13), the cultures consisted almost entirely of vimentin+ cells with rare CK19+ cells.

### Magnetic-activated CD90− cell sorting removes fibroblast-like cells

**Figure 2:**
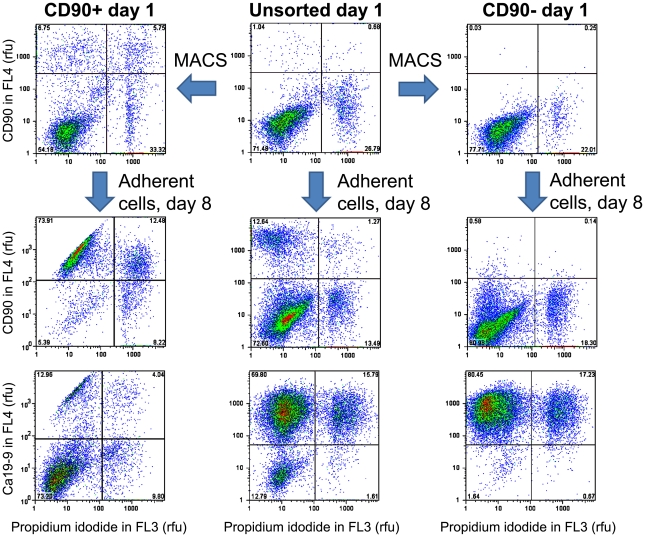
Evolution of cell populations after CD90 magnetic-activated cell sorting assessed by FACS.

A negative cell selection strategy was developed to purify human pancreatic exocrine cell cultures by removing CD90-positive cells. After 7 days of adherent culture, 34±20% of dispersed unsorted islet-depleted pancreatic cells were labelled with CD90 antibodies (Figure 2), a proportion similar to that of the spindle-shaped fibroblast-like cells. In preliminary studies, CD90-depleted cells obtained by MACS sorting on day 7 had ∼2 fold higher CK19 mRNA and ∼2.4 fold lower vimentin mRNA compared to the CD90-enriched fraction (data not shown).

**Figure 3:**
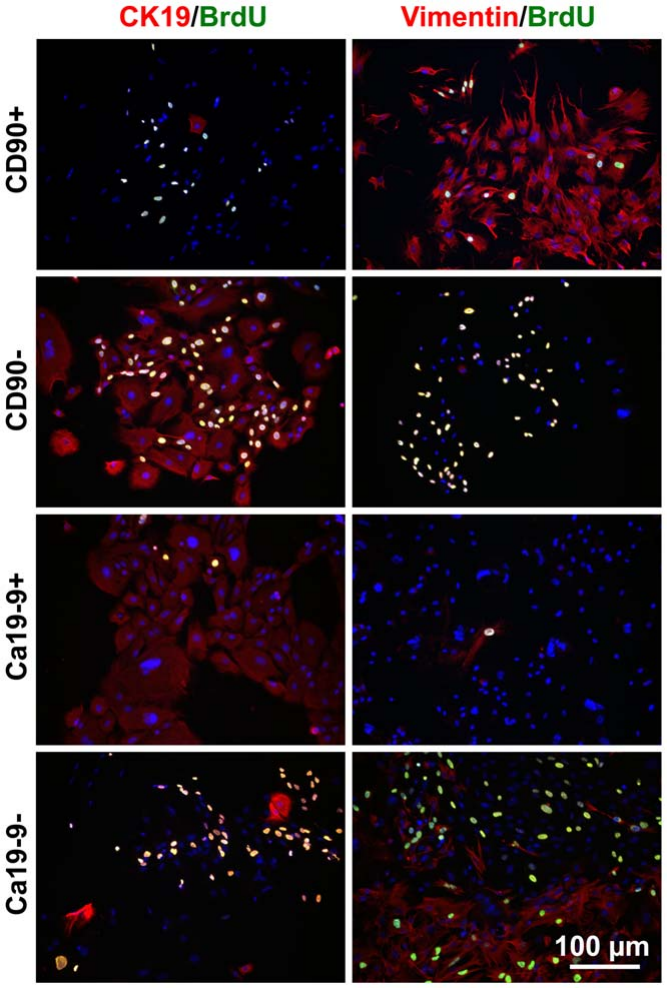
Phenotype of MACS-sorted cultures. The CD90-enriched or Ca19-9 depleted cell fractions generate cultures of spindle-shaped vimentin+ cells; while the CD90-depleted and Ca19-9 enriched fractions generate cobblestone-pattern CK19+ cells. Culture time: 8 days.

To remove fibroblast-like cells before they proliferate to become a significant fraction of the cultured cells, islet-depleted cell clusters were first plated, left to adhere overnight and sorted on day 1. Cell dispersion after overnight adhesion had the advantage of being less damaging (85±4% cell viability after dispersion) than dispersion of clusters on day 0 (71±4% viability), without any significant difference in the viable cell yield (166±72 million cells/mL initial PCV). The cell phenotypes obtained after CD90 sorting on day 1 are shown in Figures 2 and 3 as well as in Table 1. Depletion reduced the fraction of CD90+ cells on day 1 from 8±7% to 0.06±0.01%. After 7 days of culture, the unsorted cells consisted of a ∼40/60 mixture of Ca19-9+ and CD90+ cells whereas the CD90-depleted cultures consisted of >85% Ca19-9+ cells (Figure 2). On day 8, the CD90-depleted cell cultures consisted mainly of CK19+ cells growing in cobblestone patterns typical of epithelial cells, while the CD90-enriched cultures consisted mainly of spindle-shaped fibroblast-like vimentin+ cells (Figure 3). Compared to unsorted tissue, CD90 depletion doubled the fraction of CK19+ cells on day 8, similar to the CK19+ fraction obtained by enrichment of cells expressing the Ca19-9 duct surface marker. Although a higher proportion of amylase+ cells were seeded by CD90 depletion than by Ca19-9-enrichment, there were no significant differences in the cell phenotypes obtained on day 8 (Figure 3 and Table 1). When enriched amylase+ cells obtained by CD90/Ca19-9+ cell co-depletion were seeded in one experiment, the number of viable cells enumerated on day 9 was <1% of the number of CK19+ cells in CD90-depleted cultures (data not shown), indicating that most amylase+ cells do not persist until day 8 in adherent cultures.

**Table 1:** Fractions of cells obtained before and after sorting on day 1.

Cell population	Day seeded	Day analyzed	% positive cells by immunocytochemistry			% positive cells by FACS	
			Amylase	CK19	Vimentin	CD90	Ca19-9
Unsorted	0	0	61±16^b^	6 ±3	12±6	N/A	N/A
Unsorted	0	1	47±17	22±6^a^	11±4^a^	8±7^a^	54±16
CD90−	0	1	42±10	18±6^a^	8±2^a^	0.06±0.01^a^	N/A
CD90+	0	1	19±5	12±6^a^	42±18^a^	26±10^a^	N/A
Ca19-9+	0	1	16±8	51±21^a^	2±2^a^	N/A	96±2
Ca19-9−	0	1	72±14	5±2^a^	24±9^a^	N/A	14±13
Unsorted	0	8	0.6^c^	32±7	18±11	48±18	46±20
Unsorted	1	8	0.5^c^	36±10	21±5	27±9^a^	60±11^a^
CD90−	1	8	2.3^c^	59±4	10±2	0.7±0.4^a^	79±8^a^
CD90+	1	8	7.4^c^	16±3	54±21	84±3	14±1^b^
Ca19-9+	1	8	0.6^c^	55±10	7±2	0.7±0.2^b^	93±4^b^
Ca19-9−	1	8	0.5^c^	25±10	48±18	67±9^b^	4±1^b^

Note: unless otherwise indicated, the number of replicates is N = 3 pancreata.

a Number of replicate pancreata = 4.

b Number of replicate pancreata = 2.

c Number of replicate pancreata = 1.

N/A = non-available.

### Development of a screening platform to optimize duct cell expansion

**Figure 4:**
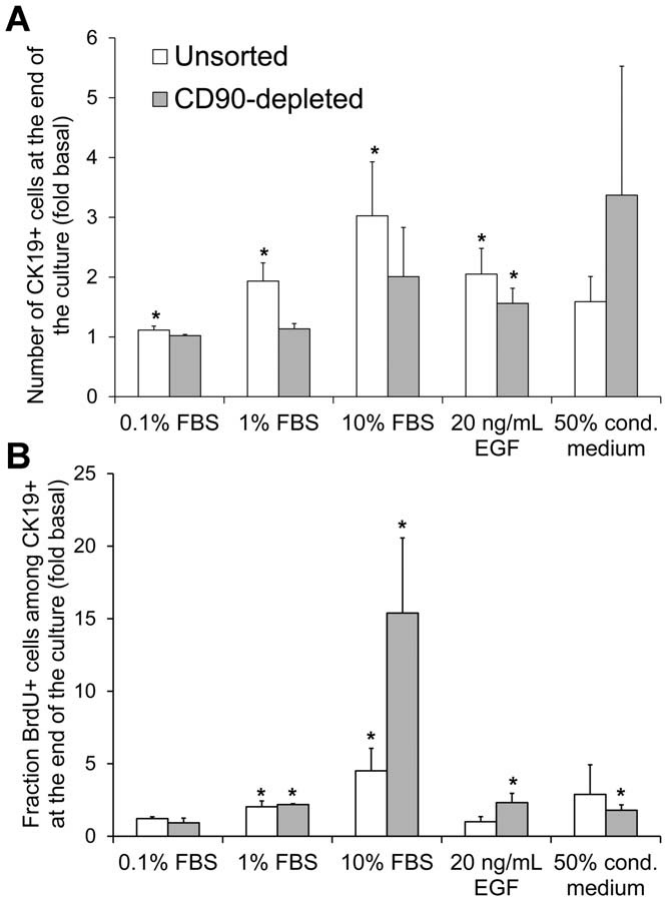
Validation of a screening platform to identify CK19+ cell mitogens. A) Number of CK19+ cells at the end of the culture normalized to the number obtained in basal serum-free medium culture. B) Fraction of CK19+ cells that incorporated BrdU during the last day of culture, normalized to BrdU incorporation in basal medium culture. Unsorted cells were seeded on day 0 while CD90-depleted cells were sorted and seeded on day 1. The data were pooled from cultures ending on day 8 (N = 5 pancreata) or day 6 (N = 3 pancreata) and normalized for each pancreas prior to calculating averages and errors. The non-normalized results obtained at the end of the cultures in basal medium were as follows: the unsorted populations contained 0.05±0.02×10^5^ CK19+ cells/cm^2^ of which 6±3% were BrdU+; the CD90-depleted populations contained 0.07±0.02×10^5^ CK19+ cells/cm^2^ of which 1.0±0.6% were BrdU+. *p<0.05 compared to basal medium.

Next we sought to identify factors capable of promoting human CK19+ cell expansion and to develop a serum-free medium for CK19+ cell culture. Cells in the negative controls were cultured in serum-free basal medium, while FBS or the reported duct mitogen EGF were added to the positive controls. Two responses after culture were quantified: the number of CK19+ cells (Figure 4A), or the BrdU incorporation by CK19+ cells (Figure 4B). The cell expansion rate varied between pancreata. To render the results between pancreata more comparable and to avoid saturation of the CK19+ cell number read-out, all cultures were ended when cells in the 10% FBS control reached confluency. The amplitude of the response to FBS was significantly higher (p = 0.005, paired t-test for the 0.1%, 1% and 10% FBS responses) for the BrdU response than for the CK19+ cell number. In addition, contrary to the CK19+ cell number, BrdU incorporation was consistently increased in the 10% FBS positive control condition for all 6 pancreata, even for the 2 pancreata where confluency (∼5000 cells/well) was reached by day 6. Another consideration addressed in these experiments was whether soluble factors secreted by the fibroblast-like CD90+ cells affected CK19+ cell expansion. The addition of 50% conditioned medium from passaged CD90-enriched cells tended to increase both the number and the BrdU incorporation of CK19+ cells. This complicated data interpretation especially for unsorted cultures because the CK19+ cell results could be indirectly influenced by factors produced by the large fraction of vimentin+ cells in these cultures (Figure 1). Based on these observations, screening experiments aiming to maximize CK19+ cell expansion were performed on CD90-depleted cell populations, using BrdU incorporation by CK19+ cells between days 5 and 6 as the measured response.

### Multifactorial screening to optimize CK19+ cell expansion

**Figure 5:**
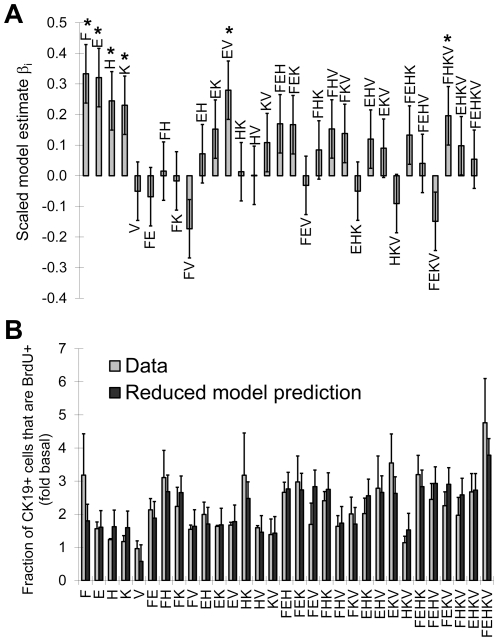
The 2^5^ full factorial effects of reported duct mitogens on CK19+ cell proliferation. The mitogens tested were bFGF (F), EGF (E), HGF (H), KGF (K) and VEGF (V) and the response measured was the fraction of BrdU+ cells among CD90-depleted CK19+ cells. A) Scaled parameter estimates of the full factorial model (*p<0.05). The “pancreas” effect, considered as a non-interacting independent variable, was non-significant and is not shown. Here, F, E, H, K and V represent their respective effects (*β_i_*) in the factorial model, divided by the *β_0_* intercept. B) Comparison between the experimental data and the reduced factorial model prediction for each combination. Here, F, E, H, K and V represent the addition of 20 ng/mL of each growth factor (e.g. the HK combination represents the addition of 20 ng/mL HGF and 20 ng/mL KGF without any other growth factors added). The values of the model predictions represent the value calculated from Equation 1 ± standard error of the model. The data were generated from N = 3 pancreata cultured for 6 days. The dataset used to generate this figure is provided as supporting information in JMP (Dataset S1) and comma-separated values (Dataset S2) formats.

**Figure 6:**
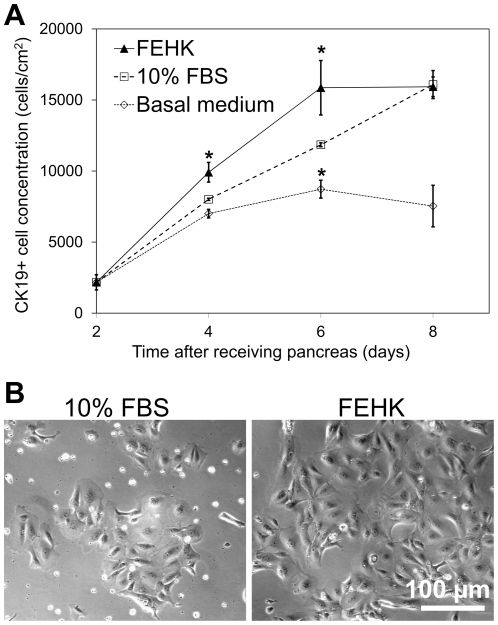
CK19+ cell expansion in medium containing bFGF, EGF, HGF and KGF compared to control media. A) Growth curve based on the number of CK19+ cells enumerated at different time points in the presence of 20 ng/mL each of bFGF, EGF, HGF and KGF or control media (*p<0.05 for two-way comparisons with the 10% FBS condition). B) Phase contrast images of the cultures taken on day 6. N = 1 pancreas with 6 replicate cultures.

The methodology developed was then applied to test the full factorial effects of five growth factors: bFGF, EGF, HGF, KGF and VEGF, using a total of 120 cultures and 3 pancreata to systematically determine the main and interaction effects of these factors. In this case, the culture time was kept constant at 6 days to simplify experimental planning and since none of the previous cultures (Figure 4) reached confluency before day 6. The high level growth factor concentrations selected for the factorial experimental design (20 ng/mL for each growth factor) were based on growth factor concentrations reported to increase duct cell expansion (Table 2.1 in reference [29]). Figure 5A shows the values of the scaled model parameters *β_i_*_,_ obtained by least squares fitting. The raw data of the full factorial experiment, including other read-outs such as the fraction number of CK19+ or vimentin+ cells is provided as supporting information in JMP (Dataset S1) and comma-separated values (Dataset S2) formats. Statistical analysis software (e.g. JMP) can be used to determine the read-outs predicted for various growth factor combinations and concentrations. The model described by Figure 5A was then reduced to include only the significant (p<0.05 in Figure 5A) parameters. No significant lack-of-fit was detected, indicating that the reduced model predicted the data within the experimental error, as can be seen in Figure 5B. The reduced model equation using scaled growth factor concentrations was:
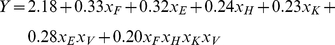
(1)where Y represents the fold increase in BrdU incorporation by CK19+ cells relative to the read-out in the presence of basal medium and *x_i_* are the growth factor concentrations scaled between −1 (0 ng/mL) and 1 (20 ng/mL). In this equation and from the scaled parameter estimates of the full factorial model (Figure 5A), it can be seen that bFGF, EGF, HGF and KGF each exerted significant positive main effects on CK19+ cell BrdU incorporation. Two significant interaction effects were also identified between EGF and VEGF, as well as bFGF, HGF, KGF and VEGF, indicating that in these combinations VEGF addition did have a positive effect. Conversely, a significant negative effect of VEGF would be expected in the absence of the other factors in these combinations (i.e. adding VEGF without also adding EGF or bFGF, HGF and KGF). Figure 5B compares the reduced model prediction to the measured fold increases in CK19+ cell BrdU incorporation for all growth factor combinations. Both the raw data and the model indicate that the combination of factors that promoted the highest CK19+ cell proliferation was the addition of all five factors (at 20 ng/mL each), but with >70% of this effect achieved by the FH (bFGF and HGF) or FK (bFGF and KGF) combinations. It should be noted that EGF and HGF were also found to have significant negative effect on the %CK19+ cells for each pancreas, respectively leading to 2±1% and 3±1% decrease in the 69±11% fraction of CK19+ cells observed if no growth factors were added (N = 3 pancreata). The effect of the bFGF, EGF, HGF and KGF (FEHK, at 20 ng/mL each) growth factor combination on CK19+ cell expansion was further examined in a time-course experiment with replicate cultures of cells from one pancreas. Figure 6A shows that the FEHK growth factor cocktail did significantly increase net CK19+ cell numbers compared to the basal medium and yielded CK19+ cell numbers equal or superior to those obtained with 10% FBS. The cultures obtained in the FEHK serum-free conditions retained the cobblestone pattern morphology typical of pancreatic duct cell cultures (Figure 6B).

## Discussion

The study and medical use of duct-like cells derived from the pancreatic tissues discarded during islet transplantation is hampered by fibroblast-like cell overgrowth and the need for an optimized defined serum-free medium for duct-like cell expansion. We present a novel method to deplete fibroblast-like cells from pancreatic tissue using CD90-based selection, as well as a serum-free high content screening platform to identify pancreatic duct mitogens.

Human duct-like cells can reportedly be maintained in culture for up to 5 weeks with fibroblast overgrowth avoided by selective cell scraping [30]. However, this would normally not be practical for larger experiments and requires human pancreatic duct dissection rather than the simpler use of the tissue discarded after islet transplantation. Pre-plating reduces but does not sufficiently eliminate contaminating fibroblast-like cells [10]. Treatment with G418 yields purified endothelial cell cultures [31], but non-specifically affects all cycling cells. Fluorescence or magnetic-activated cell sorting of CA19-9 expressing cells generates purified duct cell cultures without contribution from acinar cells [23]. However, for studies related to diabetes cellular therapy or pancreatic cancer research, the CK19+/Ca19-9+ cells arising from acinoductal transdifferentiation are of interest since they may generate insulin+ cells [32], [33] or cancer precursors [34]. In our work, CD90 was used to deplete fibroblast-like cells [20] but not acinar cells from islet-depleted pancreatic cell clusters. After one week, magnetically sorted CD90+ cells yielded fibroblast-like vimentin+ cells, while the reseeded CD90− cells yielded CK19+ cells with epithelial morphology (Figure 3) that contained virtually no CD90+ cells.

The CD90 depletion strategy was compared to the conventional Ca19-9+ cell enrichment. On day 8, there was no significant difference between the number or the fraction of CK19+ cells obtained by CD90 depletion and Ca19-9 enrichment. Yet, the initial CK19+ cell number seeded was 2.5 fold lower for the CD90− sorted cells compared to the Ca19-9+ sorted cells due to the presence of acinar cells in the CD90− sorted population. It is possible that molecules released by lysing amylase+ cells [35] or contaminating endocrine cells [36] increased the CK19+ cell proliferation in the CD90-depleted cultures by mechanisms similar to those occurring after pancreas injury [37], or that the presence of antibodies on the surface of the Ca19-9+ cells decreased their proliferation or survival. It has been suggested that acinar cells can also give rise to CK19+ cells in pancreatic cultures [33], [34], [38], [39]. However, we enumerated very few viable cells when we cultured purified acinar cells obtained by CD90 and Ca19-9 co-depletion. Similar to other reports of selective human pancreatic acinar cell death [28], [40], we did not observe any amylase+ cells co-stained with CK19, and the amylase+ cells appeared apoptotic based on their condensed nuclei [41]. These observations suggest that the CK19+ cells in the CD90-depleted cultures mainly arose from duct cells rather than transdifferentiated acinar cells.

A Cellomics-based imaging method was used to screen CK19+ cell mitogens and develop a serum-free medium adapted to these cells. CD90-depleted cells were used to avoid measuring indirect effects exerted by vimentin+ cells on the CK19+ cells. Factorial design [42], [43], [44], [45] increased the statistical power of experiments and reduced the number of runs required to elucidate factor interaction effects compared to performing a series of two-way comparisons [46]. The positive main effects of bFGF, EGF, HGF and KGF observed are consistent with their reported effects on normal [47], [48], [49], [50], [51] and neoplastic [52] ductal cells. Pancreatic knock-down of the bFGF [53] or the EGF receptor impairs embryonic ductal growth and branching morphogenesis. EGF treatment increases the proliferation of embryonic pancreatic epithelial cells *in vitro* [54] and leads to pancreatic duct hyperplasia when administered to adult pigs [55]. The secretion of KGF [56] [57], [58] and HGF [59] [60] by the embryonic pancreatic mesenchyme induces the expansion of early pancreatic epithelial progenitors [61]. The effect of VEGF was less clear, decreasing CK19+ cell proliferation by ∼70% when added alone and reducing the mitogenic effects of other factors in several 3-factor combinations, and yet increasing CK19+ cell proliferation when all factors were added together. VEGF has been shown to increase the proliferation of fetal [62] and adult [63] rat pancreatic endothelial cells *in vitro*, but did not have a significant effect on adult human islet-depleted tissue in suspension cultures [28]. The interaction effects involving VEGF that were uncovered by the factorial experiment could explain the disparity between the reported *in vitro* effects of VEGF on duct cells (i.e. because they depend on the other factors present in the culture medium). Some confusion over the effects of VEGF on duct cells *in vitro* may also arise from indirect effects of VEGF on mesenchymal cells [64], further underlining the importance of cell sorting strategies to assess the direct effects on the population of interest.

The methods developed in this work can be applied to a variety of studies involving primary human pancreatic and other tissues. In particular, CD90 depletion provides a useful tool to resolve pancreatic cell populations. For example, Ca19-9 and CD90 co-depletion could be used to obtain purified acinar cells from islet-depleted tissue. CD90-depletion could also be useful to eliminate fibroblast-like cells from dispersed human islets by removing CD90 and C19-9 expressing cells in a single step prior to NCAM+ sorting [65]. The high content duct-mitogen screening platform could be used to further optimize pancreatic CK19+ cell culture conditions, such as for testing other growth factors, basal media, alternative medium additives or culture surfaces. The factors in CD90-cell conditioned medium that cause an increased proliferation of the CK19+ cells could be identified by fractionation and mass spectrometry. Also, the bFGF, EGF, HGF and KGF-supplemented serum-free medium or other promising combinations such as bFGF and HGF or bFGF and KGF could be used in protocols requiring the serum-free expansion of pancreatic CK19+ cells. The optimal concentrations of these growth factors could be investigated by more complex multidimensional dose response studies, for example using central composite experimental design [42]. The CD90-depleted cell culture platform could be used in a variety of high content screening studies, such as to identify molecules that hinder the proliferation of pancreatic cancer cells, but not normal duct cells.

In conclusion, we describe a novel CD90 magnetic-activated cell depletion strategy to generate purified CK19+ cell cultures from primary human islet-depleted pancreatic tissue. Multifactorial high content screening of the bFGF, EGF, HGF, KGF and VEGF effects uncovered significant interactions between these factors. The growth factor combination that maximized the expansion of CK19+ cells in serum-free medium included the addition of all five factors (at 20 ng/mL concentration for each factor), whereas the combination of bFGF and HGF, or the combination of bFGF and KGF achieved ∼70% of the maximum effect. Several areas of diabetes and pancreatic cancer research could benefit from these methods to obtain purified CK19+ pancreatic cells in serum-free cultures amenable to high content screening.

## Supporting Information

Dataset S1 **Dataset used to compute the 2^5^ full factorial effects of reported duct mitogens on CK19+ cell proliferation (** **Figure 5** **).** This dataset provides the cell number, the fraction of cells expressing CK19, the fraction of cells that did not express vimentin, as well as the BrdU incorporation by the CK19+ or vimentin− cells. The data are also provided as values normalized to the read-out measured in basal medium (- - - - - condition) for each pancreas. Each read-out is provided for 40 factorial conditions, tested on the cells from 3 different pancreata. Each value provided corresponds to the average result measured for 3 replicate cultures. All cell numbers are provided for a surface area of 0.176 cm^2^ (i.e. 16 images acquired at 10× magnification on the Cellomics apparatus).
(JMP)

Dataset S2 **Dataset S1 in comma-separated values format.**
(TXT)
